# Optimized inorganic carbon regime for enhanced growth and lipid accumulation in *Chlorella vulgaris*

**DOI:** 10.1186/s13068-015-0265-4

**Published:** 2015-06-11

**Authors:** Egan J Lohman, Robert D Gardner, Todd Pedersen, Brent M Peyton, Keith E Cooksey, Robin Gerlach

**Affiliations:** Center for Biofilm Engineering and Department of Chemical and Biological Engineering, Montana State University, Bozeman, MT 59717 USA; Department of Bioproducts and Biosystems Engineering and West Central Research and Outreach Center, University of Minnesota, St. Paul, MN 55108 USA; Environmental Biotechnology Consultants, Manhattan, MT 59741 USA

**Keywords:** Triacylglycerol (TAG), Microalgae, Biodiesel, Fatty acid methyl ester (FAME), *Chlorella vulgaris*, Bicarbonate, CO_2_, Nitrogen limitation

## Abstract

**Background:**

Large-scale algal biofuel production has been limited, among other factors, by the availability of inorganic carbon in the culture medium at concentrations higher than achievable with atmospheric CO_2_. Life cycle analyses have concluded that costs associated with supplying CO_2_ to algal cultures are significant contributors to the overall energy consumption.

**Results:**

A two-phase optimal growth and lipid accumulation scenario is presented, which (1) enhances the growth rate and (2) the triacylglyceride (TAG) accumulation rate in the oleaginous Chlorophyte *Chlorella vulgaris* strain UTEX 395, by growing the organism in the presence of low concentrations of NaHCO_3_ (5 mM) and controlling the pH of the system with a periodic gas sparge of 5 % CO_2_ (*v*/*v*). Once cultures reached the desired cell densities, which can be “fine-tuned” based on initial nutrient concentrations, cultures were switched to a lipid accumulation metabolism through the addition of 50 mM NaHCO_3_. This two-phase approach increased the specific growth rate of *C. vulgaris* by 69 % compared to cultures sparged continuously with 5 % CO_2_ (*v*/*v*); further, biomass productivity (g L^−1^ day^−1^) was increased by 27 %. Total biodiesel potential [assessed as total fatty acid methyl ester (FAME) produced] was increased from 53.3 to 61 % (FAME biomass^−1^) under the optimized conditions; biodiesel productivity (g FAME L^−1^ day^−1^) was increased by 7.7 %. A bicarbonate salt screen revealed that American Chemical Society (ACS) and industrial grade NaHCO_3_ induced the highest TAG accumulation (% *w*/*w*), whereas Na_2_CO_3_ did not induce significant TAG accumulation. NH_4_HCO_3_ had a negative effect on cell health presumably due to ammonia toxicity. The raw, unrefined form of trona, NaHCO_3_∙Na_2_CO_3_ (sodium sesquicarbonate) induced TAG accumulation, albeit to a slightly lower extent than the more refined forms of sodium bicarbonate.

**Conclusions:**

The strategic addition of sodium bicarbonate was found to enhance growth and lipid accumulation rates in cultures of *C. vulgaris*, when compared to traditional culturing strategies, which rely on continuously sparging algal cultures with elevated concentrations of CO_2(g)_. This work presents a two-phased, improved photoautotrophic growth and lipid accumulation approach, which may result in an overall increase in algal biofuel productivity.

**Electronic supplementary material:**

The online version of this article (doi:10.1186/s13068-015-0265-4) contains supplementary material, which is available to authorized users.

## Background

The extraction and refinement of petroleum as a natural resource for fuel and specialty chemicals has enabled human innovation and led to unprecedented technological growth in industrialized nations; however, these technological advancements are not without cost. The current rate of producing enough crude petroleum to satisfy global demand has led to political, economic, and environmental controversy [1]. The US alone currently consumes over 18 million barrels of crude oil each day, with a significant percentage being imported from foreign sources [2]. Additionally, atmospheric concentrations of carbon dioxide have risen drastically due to the increasing combustion of fossil fuels [3, 4]. Carbon dioxide is one of the most prevalent greenhouse gases responsible for global climate change and ocean acidification. Industrial processes and the transportation sector currently contribute more than 29 billion tonnes of carbon dioxide to the atmosphere each year [3]. This is more than 400× the estimated rate of global carbon fixation by primary biota [5].

Renewable sources of energy rich, biologically produced feed stocks are needed to offset this trend. Corn, soybean, and sugar cane are the current primary sources for bioethanol and biodiesel; however these crops compete for arable land needed to produce food [6]. Algal-derived biofuels, especially biodiesel, have received increased attention in recent decades as a potential renewable energy resource [1, 7–9]. Based on life cycle analyses and conservative estimates of biodiesel yields, it has been projected that less than 5 % of currently available arable land would be required to displace 50 % of fossil fuel consumption in the USA [10]. This estimate is even more appealing when considering the fact that microalgae do not require arable land as they are not a conventional crop; rather, algal ponds require flat terrain, appropriate temperature, abundant sunlight, water, and nutrients. It should be noted, however, that non-arable land use for large-scale cultivation of microalgae will certainly impact the environment and these impacts should be evaluated.

Next to sunlight and water, the most prerequisite resource is inorganic carbon, which is necessary for photoautotrophic cell growth as well as lipid synthesis. Biodiesel, defined as fatty acid methyl ester (FAME), has similar chemical properties to traditional diesel fuel, and can be produced from energy-rich storage compounds such as triacylglycerols (TAGs), which can be synthesized by microalgae in relatively high abundance [10]. TAGs are comprised of long chain hydrocarbons (fatty acids) and a glycerol backbone, and a fundamental requirement for their synthesis is inorganic carbon availability to the organism. To realize commercial scale development of algal-derived biofuels, optimized processes for the delivery and biological uptake of inorganic carbon are important to increase productivity. This study focuses on the strategic use of bicarbonate salts to provide the necessary amounts of dissolved inorganic carbon (DIC) for increased growth and lipid production by *Chlorella vulgaris* sp. strain UTEX 395. This study demonstrates an optimized two-phase growth and lipid accumulation strategy to increase the specific growth rate and induce lipid accumulation in *C. vulgaris*. The results presented offer a promising strategy for optimizing productivity and reducing resource costs.

### Experimental design and rationale

Although microalgae will grow under atmospheric concentrations of inorganic CO_2_ (~400 ppm), improved biomass yields can be achieved by supplementing with additional inorganic carbon. It is often cited that this additional carbon source could come from industrial waste such as power plant flue gas [9, 11]. Unfortunately, studies have suggested that this process may be economically cost prohibitive [12–14]. Moreover, a recent life cycle analysis has shown that due to the inefficiency of aerating open algal ponds with CO_2_ gas, there may not be enough CO_2_ produced from waste streams required for large scale algal biofuel production [14]. This is, in part, because the kinetics of CO_2_ dissolution are slow enough that in open ponds of shallow depths, the majority of CO_2_ gas sparged into the pond can escape into the atmosphere [13, 15, 16]. The efficiency of CO_2_ transfer into aqueous solutions will, among other factors, depend on the deviation of medium conditions from equilibrium with the sparged gas (and the atmosphere), the contact time (*e.g*., depth of the water column), and the contact surface area (*e.g*., bubble size) [16–19]. Solutions with high non-carbonate alkalinity, *e.g*., solutions with high hydroxyl ion concentrations (high pH) will have a higher capacity to absorb CO_2_. In these solutions, the hydroxyl-based alkalinity will be converted into carbonate and bicarbonate alkalinity [17]. The ratio of the three different DIC species [dissolved carbon dioxide (CO_2(aq)_), bicarbonate (HCO_3_^−^), and carbonate (CO_3_^2−^)] is determined by the pH of a system, whether in equilibrium with the atmosphere or not [17, 19–21].

While research has suggested that the preferential species for microalgae is CO_2(aq)_ [22], at the optimal pH range for growth (pH 7–9), the predominant species is bicarbonate. Many microalgae have developed CO_2_ concentrating mechanisms (CCMs) which allow them to actively transport the bicarbonate anion (HCO_3_^−^) to RuBisCO and thus utilize both carbon dioxide and bicarbonate as carbon source [15, 23–31]. When HCO_3_^−^ is utilized, hydroxyl ions are released (HCO_3_^−^→CO_2_ + OH^−^). This process results in the production of hydroxyl-based alkalinity from carbonate alkalinity effectively increasing the driving force for additional CO_2_ dissolution into the medium as outlined above [17, 32].

Additionally, our previous research has revealed that the timely addition of bicarbonate to microalgae cultures induces a significant increase in TAG accumulation [33]. This “bicarbonate trigger” has been tested and optimized on various species, including Chlorophytes and diatoms [33–37]. In this study, it is compared how various bicarbonate salts induce TAG accumulation, and a two-phase, enhanced growth and lipid accumulation scenario is presented for cultures of *C. vulgaris*. By supplementing cultures with low doses of bicarbonate to first enhance the specific growth rate and secondly by adding elevated concentrations of bicarbonate in concert with medium nitrogen depletion, both growth and lipid accumulation rates were significantly increased. The description of the results and discussion is separated into two parts. The first part details the results of using various bicarbonate salts to induce lipid accumulation, while the second part presents an optimized growth and lipid accumulation scenario.

## Results and discussion

### Part 1: carbonate salt screen for lipid production

To the best of our knowledge, only ACS grade NaHCO_3_ has been used to induce TAG accumulation in microalgae [33–35, 38]. From an industrial cost perspective, a less refined and hence less expensive source of inorganic bicarbonate will be preferable. Five different bicarbonate salts and one carbonate salt (Table 1) were evaluated regarding their potential to induce TAG accumulation relative to ambient air sparged cultures using *C. vulgaris*. Briefly, batch cultures of *C. vulgaris* were grown in Bold’s basal medium until just prior to nitrogen depletion, at which time the cultures were pooled, harvested by centrifugation, and transferred into medium supplemented with inorganic carbon and deplete of any nitrogen source. Seven experimental conditions were designed to compare extractable lipid accumulation as monitored by gas chromatography-flame ionization detection (GC-FID) and total biodiesel potential via gas chromatography-mass spectroscopy (GC-MS). Extractable lipids are defined here as intracellular lipids such as TAGs, free fatty acids (FFAs), monoacylglycerides (MAGs), or diacylglycerides (DAGs), which can be liberated from lysed cells using non-polar solvents. Our previous work has shown that GC-FID analysis can be effectively used for the characterization of extractable lipids from microalgae cultures [39]. Total biodiesel potential is defined here as the mass of all fatty acids (polar and non-polar), which are quantified by transesterification into FAMEs. The values are usually expressed in weight FAME per weight biomass and are assessed based on carbon chain length and saturation via GC-MS [39]. FAMEs can originate from TAG, DAG, MAG, FFA as well as membrane and glycolipids [39, 40].

**Table 1:** C. vulgaris culture characteristics during nitrogen limited growth when supplemented with various bicarbonate salts

Treatment	Cell concentration (×10^7^ cells mL^−1^)	Cell doublings^a^	Dry weight (g L^−1^; DCW)^b^	Endpoint pH	Total chlorophyll (mg L^−1^)	Total chlorophyll per cell (pg)	Dry weight per cell (ng)
0 mM HCO_3_^−^	3.48 ± 0.17	2.47 ± 0.23	0.37 ± 0.03	8.08 ± 0.07	1.82 ± 0.08	0.052 ± 0.004	0.011 ± 0.001
50 mM ACS grade NaHCO_3_	2.15 ± 0.17^*^	1.35 ± 0.19^*^	0.58 ± 0.06^**^	9.82 ± 0.03^**^	2.00 ± 0.34	0.093 ± 0.011^**^	0.027 ± 0.001^**^
50 mM industrial grade NaHCO_3_	1.78 ± 0.13^*^	0.64 ± 0.40^*^	0.60 ± 0.02^**^	9.82 ± 0.06^**^	1.72 ± 0.18	0.097 ± 0.016^**^	0.034 ± 0.003^**^
50 mM KHCO_3_	1.56 ± 0.13^*^	0.88 ± 0.27^*^	0.54 ± 0.03^**^	9.77 ± 0.03^**^	2.01 ± 0.21	0.129 ± 0.013^**^	0.035 ± 0.003^**^
50 mM NH_4_HCO_3_	0.62 ± 0.04^*^	−0.25 ± 0.47^*^	0.15 ± 0.04^*^	9.31 ± 0.05^**^	0.31 ± 0.17^*^	0.031 ± 0.014	0.016 ± 0.005
50 mM Na_2_CO_3_	1.21 ± 0.22^*^	0.83 ± 0.15^*^	0.35 ± 0.01	9.98 ± 0.02^**^	1.37 ± 0.15^*^	0.115 ± 0.024^**^	0.029 ± 0.006^**^
25 mM NaHCO_3_∙Na_2_CO_3_	1.21 ± 0.16^*^	0.38 ± 0.42^*^	0.49 ± 0.04^**^	9.83 ± 0.03^**^	1.77 ± 0.22	0.149 ± 0.036^**^	0.041 ± 0.008^**^

Values are reported for the completion of the experiment (5.75 days) (*n* = 3)

^*^ *p* value <0.05 as determined by a two-tailed *t* test, statistically significantly lower [difference between treatment and control group (0 mM HCO_3_^−^)]

^**^ *p* value <0.05 as determined by a two-tailed *t* test, statistically significantly higher [difference between treatment and control group (0 mM HCO_3_^−^)]

^a^Cell doublings are calculated as *n* = log_2_(C_f_/C_i_); where *n* is the number of cell doublings and *C*_f_ and *C*_i_ are final and initial cell concentrations, respectively

^b^Dry cell weight (DCW) determined gravimetrically with lyophilized biomass

Six experimental groups in triplicate received the following sources of dissolved inorganic carbon (DIC, 50 mM C final concentration): (1) ACS grade NaHCO_3_, (2) industrial grade NaHCO_3_, (3) KHCO_3_, (4) NH_4_HCO_3_, (5) Na_2_CO_3_, and (6) NaHCO_3_∙Na_2_CO_3_. These treatments were compared with each other and a control, which received no additional inorganic carbon. Table 1 presents culture characteristics for each treatment at the conclusion of the experiment. As expected based on our previous work [33, 34, 41], the cultures that did not receive any bicarbonate or carbonate increased in cell concentration from 6.3 × 10^6^ cells mL^−1^ until reaching stationary growth at 3.5 × 10^7^ cells mL^−1^ (Table 1), whereas cultures supplemented with 50 mM DIC (as carbonate or bicarbonate) did not increase as much, remained stagnant, or even decreased in cell numbers (NH_4_HCO_3_). However, even though cell numbers did not increase as much in bicarbonate supplemented treatments, cell dry weights in all bicarbonate supplemented cultures, except the NH_4_HCO_3_ supplemented treatment, increased. This resulted in higher dry weights per cell in the bicarbonate- and carbonate-amended treatments compared to the control treatments (Table 1). Conversely, analysis of the concentration of chlorophyll indicates no statistical difference between the control treatments and those that received bicarbonate additions, with exception of those that received NH_4_HCO_3_ and the treatments which received carbonate. When normalized to the cell number, there was significantly more chlorophyll per cell in all salt amendments with exception to those that received NH_4_HCO_3_ (Table 1). Again, this is due to the delay or arresting of the cellular division, and this phenomenon has been well documented and is believed to be the result of a fundamental metabolic shift from growth metabolism to lipid accumulation metabolism after bicarbonate addition [33–35].

**Fig. 1:**
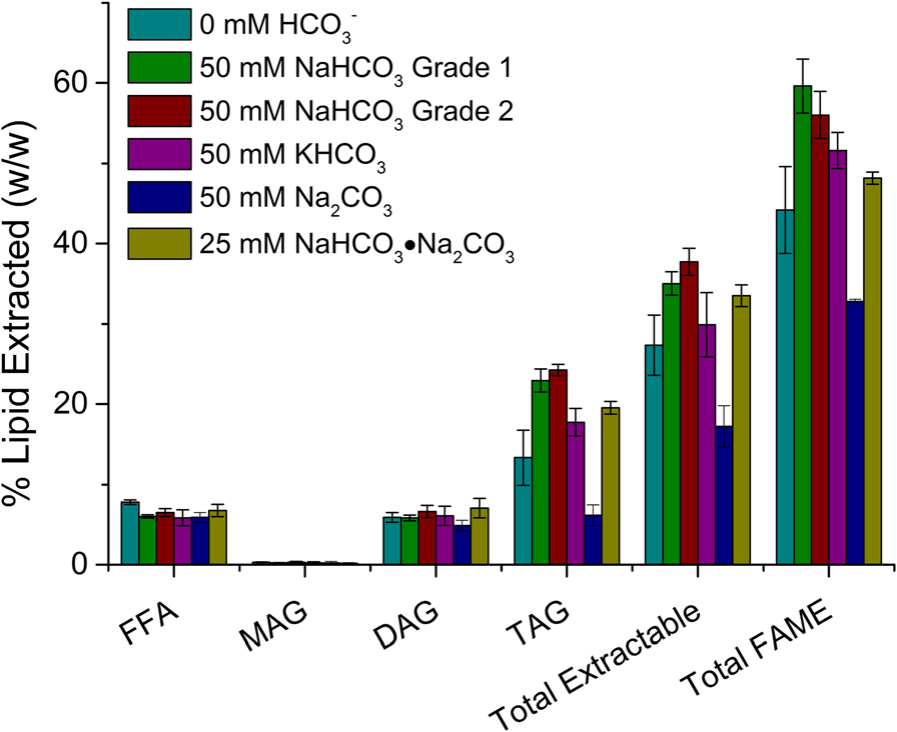
Extractable lipid class and FAME profiles for cultures of *C. vulgaris* re-suspended into medium depleted of nitrogen and supplemented with various bicarbonate salts. Final concentrations of bicarbonate salts per experimental condition: 0 mM HCO_3_^−^ (control), 50 mM ACS grade NaHCO_3_ (grade 1), 50 mM industrial grade NaHCO_3_ (grade 2), 50 mM KHCO_3_, 50 mM Na_2_CO_3_, and 25 mM NaHCO_3_∙Na_2_CO_3_ (25 mM of sesquicarbonate was used to provide equimolar carbon). Values are reported for the completion of the experiment (*n* = 3). All values expressed as weight percent (% weight extractable lipid or weight FAME/weight biomass)

Figure 1 presents the extractable lipids (FFA, MAG, DAG, TAG) and in situ transesterified FAMEs for each culture after maximum TAG accumulation (5.75 days of N-limited culturing) [39]. Cultures supplemented with 50 mM ACS or industrial grade bicarbonate accumulated the most TAG (22.9 ± 1.5 and 24.3 ± 0.7 % *w*/*w*, respectively), as compared to the cultures which did not receive any additional DIC (13.3 ± 3.4 % *w*/*w*). Cultures supplemented with 50 mM KHCO_3_ or 25 mM Na_2_CO_3_·NaHCO_3_ also accumulated a significant amount of TAG (17.7 ± 1.7 and 19.5 ± 0.797 % *w*/*w*, respectively), albeit less than cultures supplemented with ACS or industrial grade NaHCO_3_ (Fig. 1). Sodium sesquicarbonate is the raw material (*i.e*., trona) used for the production of the more refined grades of carbonates/bicarbonates. Trona typically contains high concentrations of silicates and other compounds and is likely one of the least expensive forms of bicarbonate available.

**Fig. 2:**
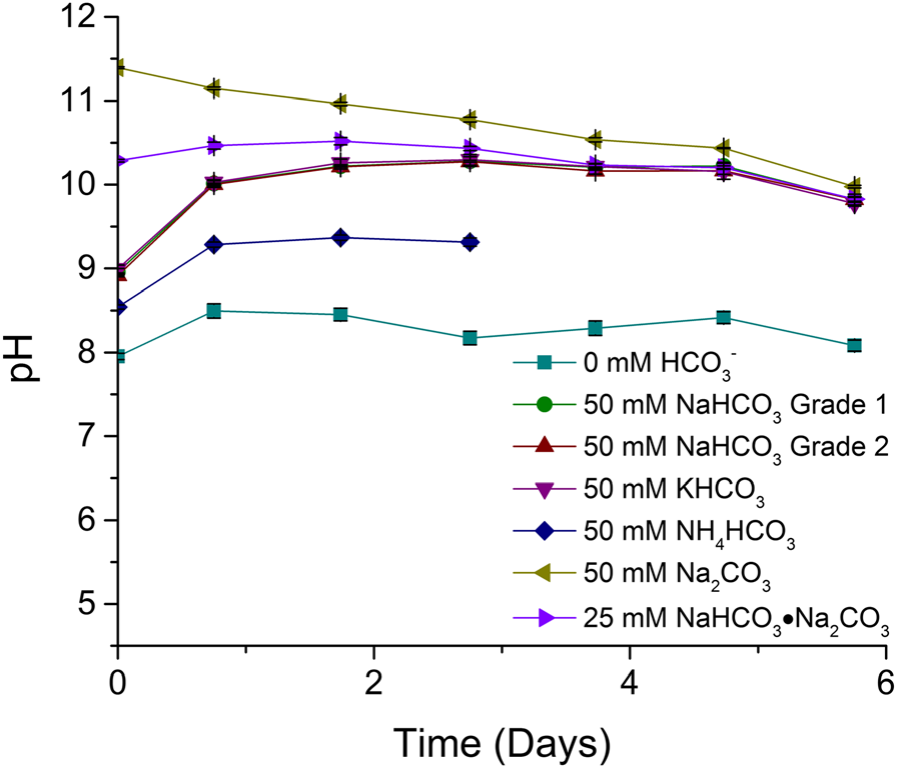
pH for cultures of *C. vulgaris* re-suspended into medium depleted of nitrogen and supplemented with various bicarbonate salts. Final concentrations of bicarbonate salts per experimental condition: 0 mM HCO_3_^−^ (control), 50 mM ACS grade NaHCO_3_ (grade 1), 50 mM industrial grade NaHCO_3_ (grade 2), 50 mM KHCO_3_, 50 mM NH_4_HCO_3_, 50 mM Na_2_CO_3_, and 25 mM NaHCO_3_∙Na_2_CO_3_ (25 mM of sesquicarbonate was used to provide equimolar carbon) (*n* = 3)

Cultures treated with 50 mM NH_4_HCO_3_ did not accumulate TAG (data not shown), declined in cell concentration after 2 days of incubation (Additional file 1) and reached cell concentrations and cell dry weights significantly lower than all the other cultures at the end of the experiment (Table 1). The final pH of the NH_4_HCO_3_ amended system was approximately 9.3 (Fig. 2, Table 1), which is equal to the pKa for the NH_4_^+^/NH_3_ equilibrium, indicating that high concentrations of NH_3_ were present. The decline in cell concentration is presumably due to the toxic effects of ammonia, which has been shown previously to inhibit photosynthesis in microalgal cultures [42].

Finally, cultures supplemented with 50 mM Na_2_CO_3_ accumulated a significantly lower percentage of TAG (6.2 ± 1.3 % *w*/*w*) compared to all other treatments (Fig. 1, Table 2). There are no known metabolic pathways through which *C. vulgaris* can assimilate CO_3_^2−^ into biomass and at the initial pH 11.4 (Fig. 2) virtually all of the dissolved inorganic carbon would have been present as carbonate (the pKa for the HCO_3_^−^/CO_3_^2−^ equilibrium is approximately at pH 10.3). However, throughout the experiment, the pH continuously decreased and reached a final pH of 9.98 (Fig. 2, Table 1); thereby, speciation was shifted to predominantly bicarbonate which became bioavailable to the alga. It is presumed that the higher sodium concentration in the CO_3_^2−^ amended systems may also have influenced lipid metabolism in *C. vulgaris*. However, Gardner et al. (2013) showed that the addition of up to 50 mM sodium ions did not have a negative effect on the lipid accumulation activity of a *Scenedesmus* sp. [41]. This effect will have to be investigated further in the future for *C. vulgaris*.

**Table 2:** FAME profiles of C. vulgaris when supplemented with various bicarbonate salts just prior to nitrogen depletion

Treatment	C16:0	C16:1	C18:0	C18:1	C18:2	C18:3	Other^a^	Total biodiesel potential (%)^b^	Potential biodiesel productivity (g L^−1^ day^−1^)^b^
0 mM HCO_3_^−^	7.51 ± 0.69	1.59 ± 0.16	4.72 ± 0.5	16.82 ± 2.97	3.92 ± 0.43	9.3 ± 0.75	0.4 ± 0.1	44.19 ± 5.42	0.029 ± 0.004
50 mM ACS grade NaHCO_3_	7.78 ± 0.46	2.33 ± 0.18	2.46 ± 0.14	31.82 ± 1.73	5.61 ± 0.35	9.06 ± 0.43	0.58 ± 0.06	59.63 ± 3.34^*^	0.061 ± 0.008^*^
50 mM industrial grade NaHCO_3_	7.38 ± 0.37	2.11 ± 0.05	2.28 ± 0.18	30.06 ± 1.39	5.16 ± 0.13	8.43 ± 0.74	0.61 ± 0.05	56.04 ± 2.9^*^	0.058 ± 0.001^*^
50 mM KHCO_3_	6.8 ± 0.22	1.79 ± 0.05	2.64 ± 1.11	27 ± 2.08	5.15 ± 0.07	7.74 ± 0.3	0.54 ± 0.13	51.62 ± 2.25^*^	0.048 ± 0.012^*^
50 mM Na_2_CO_3_	5.12 ± 0.11	1.26 ± 0.03	2.56 ± 0.04	14.47 ± 0.12	3.4 ± 0.02	5.84 ± 0.05	0.2 ± 0.05	32.82 ± 0.28	0.02 ± 0.001
25 mM NaHCO_3_∙Na_2_CO_3_	6.69 ± 0.08	1.84 ± 0.03	3.82 ± 0.09	23 ± 0.62	4.67 ± 0.09	7.72 ± 0.12	0.47 ± 0.08	53.33 ± 1.35^*^	0.041 ± 0.003^*^

Values are reported for the completion of the experiment (5.75 days) (*n* = 3). All values expressed as weight percent (% weight FAME/weight biomass) unless indicated otherwise

^*^ *p* value <0.05 as determined by a two-tailed *t* test, statistically significant higher [difference between treatment and control group (0 mM HCO_3_^−^)]

^a^Sum of other compounds detected

^b^Total FAMEs

Free fatty acid, MAG and DAG contents were statistically the same in all treatments (6.5 ± 0.3, 0.24 ± 0.06, 6.1 ± 0.34 % *w*/*w*, respectively, Fig. 1); thus, the total extractable lipid content (sum of FFA, MAG, DAG, and TAG) for each culture followed the same trend as the TAG content per cell. Total biodiesel potential (as % *w*/*w* FAME) and potential biodiesel productivity (as g L^−1^ day^−1^ FAME) [43] mirrored the trend of the TAG contents. Cultures treated with 50 mM ACS and industrial grade NaHCO_3_ accumulated the most FAME at 59.6 ± 3.3 and 56.0 ± 2.9 % *w*/*w* and had the highest potential biodiesel productivity with 0.061 ± 0.008 and 0.058 ± 0.001 g L^−1^ day^−1^, respectively (Table 2). FAME profiles are further expanded upon in Table 2, which presents a number of FAMEs separated by carbon chain length and saturation. Overall, *C. vulgaris* primarily synthesized C_18_ fatty acids, specifically mono-unsaturated C_18_ as reported previously [38, 39, 44]. The cultures which did not receive additional inorganic carbon, however, produced only approximately half (16.8 ± 3 % *w*/*w*) as much C18:1 fatty acids compared to the treatment which received 50 mM ACS grade NaHCO_3_ (31.8 ± 1.7 % *w*/*w*) or the industrial grade NaHCO_3_ (30.1 ± 1.4 % *w*/*w*). This indicates that a large fraction of the C18:1fatty acids were synthesized as a result of elevated concentrations of DIC (Table 2). Interestingly, the cultures which did not receive additional inorganic carbon produced equivalent concentrations of fully saturated C16:0 fatty acids and poly-unsaturated C18:3 fatty acids compared to treatments which received sodium bicarbonate (Table 2).

These results are consistent with previous reports describing changes in the fatty acid profiles of C. *vulgaris* UTEX 395 when cultured under nitrogen deprivation [44, 45]. For example, Guarnieri et al. (2011) reported a 9-fold increase in C18:1 fatty acid when UTEX 395 was cultured in nitrogen-free medium. Here, the addition of sodium bicarbonate resulted in a 2-fold increase in C18:1 fatty acid content based on cell dry weight over the bicarbonate-free control, which suggests that bicarbonate can enhance fatty acid synthesis in concert with nitrogen depletion.

The results from evaluating the effect of (bi)carbonate salts on lipid accumulation provided evidence that the timely addition of bicarbonate, when coupled with nitrogen depletion, can induce significant lipid accumulation and promote the preferential production of C18:1 fatty acid in *C. vulgaris*. Not only was the lipid content per biomass (biodiesel potential in % *w*/*w*) higher in the bicarbonate triggered cells, but due to the higher dry weight for the bicarbonate triggered cells, the potential biodiesel productivity (g FAME L^−1^d^−1^) was also higher in the bicarbonate-triggered cultures. However, as noted, the bicarbonate addition affects cell division and actually decreases the number of newly produced cells during the lipid accumulation phase. Therefore, in part 2 of this study, a strategy was developed that allowed for faster biomass production through the addition of small amounts of bicarbonate during the initial culturing before lipid accumulation was triggered. Implementation of such an optimized strategy may further improve lipid productivity of algal cultures.

### Part 2: optimized growth and lipid accumulation

Part 1 of this manuscript described that the lipid content (in % lipid per biomass) can be increased through bicarbonate supplementation of *C. vulgaris* cultures. However, cell replication was largely arrested at the time of bicarbonate addition (Table 1). Based on these as well as previously reported observations [33–35], it was hypothesized that enhanced cell growth and thus higher culture productivity might be achievable by adding low concentrations of bicarbonate before inducing lipid production using a second addition of bicarbonate salt at a higher concentration near nitrogen depletion.

Industrial and laboratory systems often use CO_2_ (1–5 % up to 100 % *v*/*v*) to control the pH of algal cultures between 8.0 and 8.7 [44, 46, 47]. This process can be automated via pH controllers, which initiate a CO_2_ gas sparge when the pH values in the culture medium reach undesirably high levels and discontinue the addition of CO_2_ once the pH has been lowered sufficiently.

In an attempt to increase biomass production prior to inducing lipid accumulation, 5 mM ACS grade NaHCO_3_ (final concentration at inoculation) was added to the medium at the beginning of cultivation and a pH controller maintained the medium pH between 8.4 and 8.7 by automatically sparging the system with 5 % CO_2_ (*v*/*v*) when the pH reached 8.7 and turning off when the pH had declined to 8.4. These cultures were also supplemented with 50 mM ACS grade NaHCO_3_ at day 4, just prior to nitrogen depletion, to induce TAG accumulation. This optimized growth and lipid accumulation strategy (optimized scenario) was compared to the following four culture conditions: (1) cultures sparged with atmospheric air only, (2) cultures sparged with atmospheric air and the medium supplemented with 5 mM NaHCO_3_ prior to inoculation, (3) cultures sparged with atmospheric air and pH regulated with 5 % CO_2_ (*v*/*v*) during the daylight hours to maintain a pH range of 8.4–8.7, and (4) cultures aerated with 5 % CO_2_ (*v*/*v*) continuously during the daylight hours. In this way, each inorganic carbon regime (*i.e*., 5 mM NaHCO_3_, 5 % CO_2_, pH regulated, and atmospheric air only) was evaluated individually, and the optimized scenario could be compared to those individual systems. This part of the study was not intended to be a full factorial design study, but rather synthesized the knowledge of previous work (reported here and elsewhere) to devise a strategy for improved growth *and* lipid accumulation without the need for using high concentration and possibly high-purity sources of CO_2_ for algal growth and lipid accumulation.

**Fig. 3:**
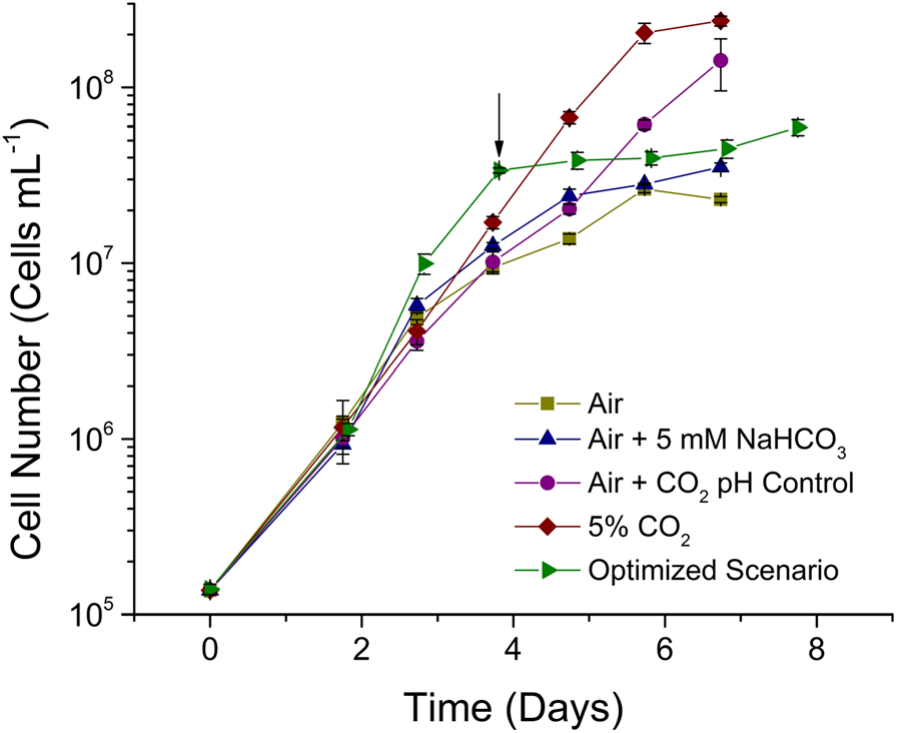
Growth (cells mL^−1^) of cultures of *C. vulgaris* cultured under various inorganic carbon regimes. (*Square*) Continuous sparge of atmospheric air, (*triangle*) continuous sparge of atmospheric air and supplemented with 5 mM NaHCO_3_ at inoculation, (*circle*) continuous sparge of atmospheric air supplemented periodically with 5 % CO_2_ (*v*/*v*) to maintain pH between 8.4 and 8.7, (*diamond*) continuous sparge of atmospheric air supplemented with 5 % CO_2_ (*v*/*v*) during daytime hours, and (*right pointing triangle*) the optimized scenario of a continuous sparge of atmospheric air supplemented periodically with 5 % CO_2_ (*v*/*v*) to maintain pH between 8.4 and 8.7 and an initial addition of 5 mM NaHCO_3_ at inoculation plus an additional 50 mM NaHCO_3_ just prior to nitrogen depletion to stimulate TAG accumulation (*n* = 3). *Arrow* indicates time of 50 mM NaHCO_3_ addition just prior to nitrogen depletion of the culture medium

The initial addition of 5 mM bicarbonate increased the average specific growth rate and biomass productivity (in g CDW L^−1^ day^−1^) for the optimized scenario by 69 and 27 %, respectively, compared to the other treatments (Fig. 3, Table 3). Furthermore, the addition of 50 mM bicarbonate to the optimized scenario on day 4 resulted in the expected cessation of cellular replication (Fig. 3, black arrow and Table 3) and increased lipid accumulation (discussed below) [33–35].

**Table 3:** C. vulgaris culturing characteristics when grown under various inorganic carbon regimes

Treatment	Cell concentration (×10^7^ cells mL^−1^)	Specific growth rate (*μ*_max_ day^−1^)	Biomass productivity (g L^−1^ day^−1^; DCW)^a^	Maximum chlorophyll (mg L^−1^)
Air only	2.31 ± 0.09^*^	0.61 ± 0.09^*^	0.04 ± 0.00^*^	3.52 ± 1.0^*^
Air + 5 mM NaHCO_3_	3.53 ± 0.2^*^	0.82 ± 0.02^*^	0.06 ± 0.01^*^	5.72 ± 1.0^*^
Air + 5 % (*v*/*v*) CO_2_ pH control	14.21 ± 4.67^**^	0.76 ± 0.05^*^	0.11 ± 0.02^*^	8.01 ± 1.23^*^
5 % (*v*/*v*) CO_2_ continuous	23.92 ± 1.55^**^	1.02 ± 0.02^*^	0.11 ± 0.01^*^	8.13 ± 1.46^*^
Optimized scenario	5.93 ± 0.61^b^	1.72 ± 0.06	0.14 ± 0.00	10.7 ± 0.84

Experimental conditions: (1) continuous sparge of atmospheric air, (2) continuous sparge of atmospheric air and supplemented with 5 mM NaHCO_3_ at inoculation, (3) continuous sparge of atmospheric air supplemented periodically with 5 % CO_2_ (*v*/*v*) to maintain pH between 8.4 and 8.7, (4) continuous sparge of atmospheric air supplemented with 5 % CO_2_ (*v*/*v*) during daytime hours, and (5) the optimized scenario of a continuous sparge of atmospheric air supplemented periodically with 5 % CO_2_ (*v*/*v*) to maintain pH between 8.4 and 8.7 and an initial addition of 5 mM NaHCO_3_ at inoculation plus an additional 50 mM NaHCO_3_ just prior to nitrogen depletion to stimulate TAG accumulation (*n* = 3). All growth yields are calculated for the exponential growth phase (*i.e*., from inoculation until depletion of nitrogen)

^*^ *p* value <0.05 as determined by a two-tailed *t* test, statistically significantly lower (difference between treatment and optimized scenario)

^**^ *p* value <0.05 as determined by a two-tailed *t* test, statistically significantly higher (difference between treatment and optimized scenario)

^a^Dry cell weight (DCW) determined gravimetrically with lyophilized biomass

^b^50 mM NaHCO_3_ addition results in cessation of cellular division

**Fig. 4:**
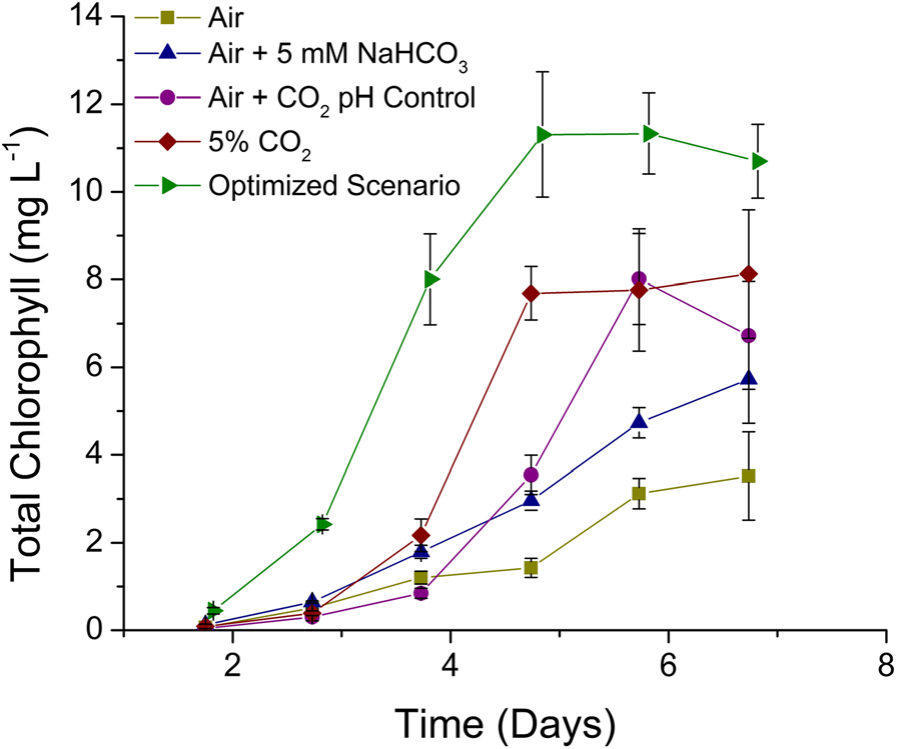
Total chlorophyll concentration (mg L^−1^) for cultures of *C. vulgaris* cultured under various inorganic carbon regimes. (*Square*) Continuous sparge of atmospheric air, (*triangle*) continuous sparge of atmospheric air and supplemented with 5 mM NaHCO_3_ at inoculation, (*circle*) continuous sparge of atmospheric air supplemented periodically with 5 % CO_2_ (*v*/*v*) to maintain pH between 8.4 and 8.7, (*diamond*) continuous sparge of atmospheric air supplemented with 5 % CO_2_ (*v*/*v*) during daytime hours, (*right pointing triangle*) the optimized scenario of a continuous sparge of atmospheric air supplemented periodically with 5 % CO_2_ (*v*/*v*) to maintain pH between 8.4 and 8.7 and an initial addition of 5 mM NaHCO_3_ at inoculation plus an additional 50 mM NaHCO_3_ just prior to nitrogen depletion to stimulate TAG accumulation (*n* = 3)

The total chlorophyll content of the cultures was monitored throughout the experiment to estimate the photosynthetic potential of each culture (Fig. 4). The optimized scenario exhibited an early increase in chlorophyll concentration, as compared to the other cultures, indicating a higher degree of culture health and photosynthetic potential during the exponential growth phase. Similarly, the concentration of chlorophyll was higher in the optimized scenario during the nitrogen limitation induced lipid accumulation phase (after day 4). This increased chlorophyll content along with the higher carbon fixation rates indicates a higher photosynthetic performance of the alga when cultured under the optimized scenario; however, additional photosynthetic parameters will need to be monitored in the future to verify this interpretation.

**Fig. 5:**
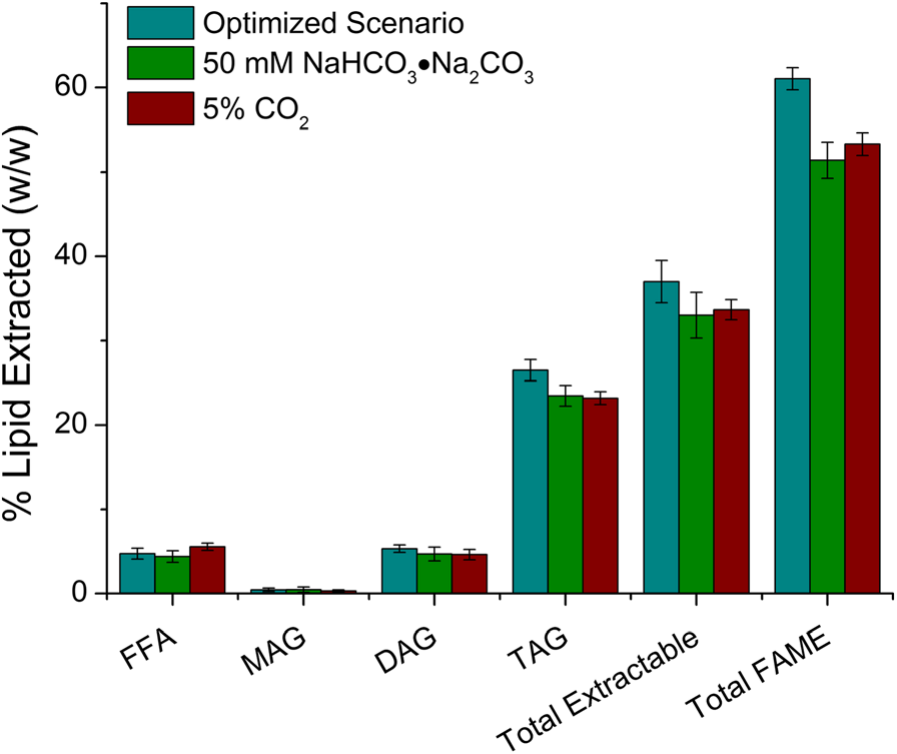
Extractable lipid class and FAME profiles for cultures of *C. vulgaris* when cultured under various inorganic carbon regimes. Experimental conditions: (1) the optimized scenario of a continuous sparge of atmospheric air supplemented periodically with 5 % CO_2_ (*v*/*v*) to maintain pH between 8.4 and 8.7 and an initial addition of 5 mM NaHCO_3_ at inoculation plus an additional 50 mM of ACS grade NaHCO_3_ just prior to nitrogen depletion to stimulate TAG accumulation. (2) The same culture conditions as scenario 1 listed above, except TAG accumulation was induced by adding 25 mM NaHCO_3_∙Na_2_CO_3_ (sesquicarbonate). (3) Continuous sparge of atmospheric air supplemented with 5 % CO_2_ (*v*/*v*) during daytime hours. Values are reported for the completion of the experiment (*n* = 3). All values expressed as weight percent (% weight extractable lipid or weight FAME/weight biomass)

To evaluate the extent of lipid accumulation, lipid profiles were compared for cultures sparged continuously with 5 % CO_2_, during the daylight hours, against cultures grown under the optimized scenario (Fig. 5, Table 4). Additionally, to assess how cultures grown under the optimized scenario would perform when using a lower grade of bicarbonate, a third set of cultures was supplemented with 50 mM NaHCO_3_∙Na_2_CO_3_ (sesquicarbonate), instead of 50 mM ACS grade NaHCO_3_, to induce lipid accumulation. Lipid profile data for cultures grown on only atmospheric air (with and without 5 mM NaHCO_3_) or air with pH regulated with 5 % CO_2_ (*v*/*v*) have been omitted for clarity because these systems did not accumulate lipid to a larger degree than the cultures which received 5 % CO_2_ continuously.

**Table 4:** C. vulgaris lipid characteristics for cultures grown under various inorganic carbon regimes

Treatment	C16:0^a^	C16:1^a^	C18:0^a^	C18:1^a^	C18:2^a^	C18:3^a^	Other^b^	Total biodiesel potential (%)^c^	Potential biodiesel productivity (g L^−1^ day^−1^)^c^
Optimized Scenario	10.66 ± 0.3	2.76 ± 0.03	5.17 ± 0.09	24.42 ± 0.44	7.17 ± 0.14	10.21 ± 0.33	0.64 ± 0.06	61.04 ± 1.31	0.098 ± 0.002
50 mM NaHCO_3_∙Na_2_CO_3_	9.27 ± 0.34	2.64 ± 0.1	3.97 ± 0.11	19.85 ± 1.2	6.56 ± 0.18	8.45 ± 0.15	0.68 ± 0.09	51.41 ± 2.14^*^	0.07 ± 0.005^*^
5 % (*v*/*v*) CO_2_ Continuous	9.45 ± 0.7	1.85 ± 0.14	2.36 ± 0.2	26.59 ± 2.1	4.58 ± 0.4	7.88 ± 0.76	0.63 ± 0.12	53.33 ± 1.35^*^	0.091 ± 0.005^*^

Experimental conditions: (1) the optimized scenario of a continuous sparge of atmospheric air supplemented periodically with 5 % CO_2_ (*v*/*v*) to maintain pH between 8.4 and 8.7 and an initial addition of 5 mM NaHCO_3_ at inoculation plus an additional 50 mM of ACS grade NaHCO_3_ just prior to nitrogen depletion to stimulate TAG accumulation. (2) The same culture conditions as scenario 1 listed above, except TAG accumulation was induced by adding 25 mM NaHCO_3_∙Na_2_CO_3_ (sesquicarbonate). (3) Continuous sparge of atmospheric air supplemented with 5 % CO_2_ (*v*/*v*) during daytime hours (*n* = 3). Biodiesel productivity is calculated for the stationary growth phase (*e.g*., from depletion of nitrogen until termination of experiment)

^*^ *p* value <0.05 as determined by a two-tailed *t* test, statistically significantly lower (difference between treatment and optimized scenario)

^a^All values expressed as weight percent (% weight FAME/weight biomass)

^b^Sum of other compounds detected

^c^Total FAMEs

Figure 5 presents extractable lipid profiles for the three treatments that accumulated significant lipids as well as total extractable lipid (sum of FFA, MAG, DAG, and TAG) and total biodiesel potential (% FAME *w*/*w* biomass). No statistical difference was observed in TAG content between cultures sparged with 5 % CO_2_ (23.2 ± 0.77 % *w*/*w*) and cultures supplemented with 50 mM NaHCO_3_∙Na_2_CO_3_ (23.5 ± 1.5 % *w*/*w*). Cultures supplemented with 50 mM ACS grade NaHCO_3_ on day 4 (Optimized Scenario) increased in TAG content by 3.3 % (*w*/*w*) over the 5 % CO_2_ control (*t* test *p* < 0.05). Free fatty acid, MAG and DAG contents were statistically equivalent between all three cultures. Total biodiesel potential (% FAME biomass^−1^) was increased from 53.3 ± 1.34 (% *w*/*w*) (5 % CO_2_ continuously) to 61 ± 1.3 (% *w*/*w*) under the optimized scenario (Fig. 5), and the averaged biodiesel productivity over the 7-day culturing time was increased by 7.7 % (Table 4). The increase in total biodiesel potential is partially attributed to increased concentrations of TAG in the optimized scenario; however, a portion also appears to have been derived from membrane-bound lipids or glycolipids. Table 4 presents FAME profiles for each of the three cultures, separated by carbon chain length and saturation. As observed in the first part of this work, cells supplemented with ACS grade sodium bicarbonate accumulated slightly, yet statistically significantly (*t* test, *p* < 0.05) more of each fatty acid (in % weight FAME/weight biomass) except for the mono-unsaturated C_18_ FAMEs, which reached the highest concentration in cultures grown on 5 % CO_2_, albeit not significantly higher (*t* test, *p* = 0.15). As discussed in part 1, changes in lipid profiles during nitrogen starvation or bicarbonate addition have been reported previously and the observations described here largely agree with those reports [38, 39, 44].

## Conclusions

A study was conducted comparing the influence of various bicarbonate salts on growth and lipid accumulation in the model Chlorophyte *Chlorella vulgaris* sp. strain UTEX 395. An optimized, two-phase enhanced growth and lipid accumulation scenario was developed, which uses strategic additions of sodium bicarbonate to enhance growth rates and lipid accumulation rates in cultures of *C. vulgaris*, as compared to traditional growth regimes which usually supply elevated concentrations of CO_2(g)_ as the sole inorganic carbon substrate. From an industrial perspective, the transport, storage, and delivery of gaseous CO_2_ has been suggested to be costly for large-scale algal production [7, 48–50]. However, competitive growth rates in microalgae cultures may only be achievable when elevated concentrations of dissolved inorganic carbon are present in the medium. By supplementing cultures with low doses of bicarbonate to first enhance the specific growth rate, and secondly by adding elevated concentrations of bicarbonate in concert with medium nitrogen depletion, both growth and lipid accumulation rates were increased for the optimized scenario by 69 and 27 %, respectively, above cultures which received 5 % CO_2_ continuously. In addition, it was found that the raw, unprocessed form of bicarbonate (sesquicarbonate) could be an adequate source of inorganic carbon for both enhanced growth and lipid accumulation in cultures of *C. vulgaris*. These data indicate that the type and strategy (*e.g*., timing, concentration, and purity) of inorganic carbon addition may have a significant influence on lipid production in *C. vulgaris*.

In summary, the strategies presented here could contribute towards a potentially cost-competitive approach to optimizing dissolved inorganic carbon supply in algal biofuel production through alkalinity control via the strategic addition of bicarbonate salts. The feasibility of this technology might be location specific and should be assessed using a techno-economic analysis to compare costs associated with bicarbonate addition compared to gaseous CO_2_ addition. Beyond further optimization of inorganic carbon supply, additional optimization can perceivably be achieved via improved strain selection and optimized light parameters (*i.e*., photosynthetic flux available to the algae due to culture density). Much of this work is in progress in our laboratories and elsewhere.

## Methods

### Strain and culturing conditions

*Chlorella vulgaris* sp. strain UTEX 395 (University of Texas at Austin) was cultured on Bold’s basal medium [51] with pH adjusted to 8.7 prior to autoclaving. Cultures were either grown in batch airlift tube reactors or baffled flasks, depending on the experimental conditions, as outlined below.

### Part 1: bicarbonate salt study

Cultures of *C. vulgaris* were grown in 100 mL of medium in a 250-mL baffled shaker flask (Fisher Scientific, Palatine, IL) until just prior to medium nitrogen depletion. Temperature was maintained at 24 °C ± 1 °C. Light (200 μmol photons m^−2^ s^−1^) was maintained on a 14:10 L/D cycle using a light incubator (Percival Scientific, Inc., Perry, IA). The biomass was concentrated via centrifugation at 4800×*g* and 4 °C for 10 min (Thermo Scientific, Sorvall Legend XTR, Waltham, MA). Biomass pellets were re-suspended in 100 mL of Bold’s basal medium depleted of nitrogen and amended with one of the following bicarbonate salts (final concentrations): 0 mM HCO_3_^−^ (control), 50 mM ACS grade NaHCO_3_, 50 mM ACS grade Na_2_CO_3_ (both from Sigma-Aldrich, St. Louis MO), 50 mM industrial grade NaHCO_3_, 50 mM NH_4_HCO_3_, 50 mM KHCO_3_, and 25 mM natural grade NaHCO_3_∙Na_2_CO_3_ (sesquicarbonate) (all from Church & Dwight Co., Inc.; Princeton, NJ). All studies were conducted in triplicate.

### Part 2: inorganic carbon growth and lipid accumulation study

Experiments were conducted in triplicate batch cultures using 70 × 500 mm glass tubes containing 1.2 L medium submersed in a water bath to control temperature at 24 °C ± 1 °C. Rubber stoppers, containing ports for aeration and sampling, were used to seal the tubes. Light (400 μmol photons m^−2^ s^−1^) was maintained on a 14:10 L/D cycle using a light bank containing T5 fluorescent tubes. Aeration (400 mL min^−1^) was supplied by humidified compressed air (supplemented with 5 % CO_2_ (*v*/*v*) for high CO_2_ or pH-controlled conditions during daylight hours) and controlled using individual rotameters for each bioreactor (Cole-Parmer, USA). ACS grade sodium bicarbonate was used in all experiments involving bicarbonate addition (Sigma-Aldrich, St. Louis MO), and natural grade NaHCO_3_∙Na_2_CO_3_ (Church & Dwight, Princeton, NJ) was used in all experiments involving sesquicarbonate.

### Culture analysis

Cultures were checked for bacterial contamination by streaking culture subsamples onto Bold’s basal medium agar supplemented with 0.05 % yeast extract and 0.05 % glucose. No growth of colonies was detected after 7 days of incubation at room temperature in the dark. Cell concentrations were determined using an optical hemacytometer with a minimum of 400 cells counted per sample for statistical reliability. Dry cell weights (DCWs) were determined by transferring 50 mL of culture into a pre-weighed 50 mL centrifuge tube (Fisher Scientific, Palatine, IL), followed by centrifugation at 4800×*g* at 4 °C for 10 min (Thermo Scientific, Sorvall Legend XTR, Waltham, MA). The concentrated biomass was rinsed with deionized H_2_O (diH_2_O), 18 MΩ, to remove media salts and excess bicarbonate, before centrifuging again. Remaining algal pellets were frozen and lyophilized (Labconco lyophilizer, Kansas City, MO) for 48 h. DCWs were calculated by subtracting the weight of the biomass-free centrifuge tube from the weight of the centrifuge tube with lyophilized biomass.

### Analysis of media components

Medium pH was measured using a standard bench top pH meter. Nitrate concentrations were measured using ion chromatography (Dionex, Sunnyvale, CA), respectively, using previously described protocols [34].

### Dissolved inorganic carbon analysis

Dissolved inorganic carbon (DIC, sum of dissolved carbon dioxide, bicarbonate, and carbonate) in the medium was measured using previously described protocols [34]. Briefly, 8 mL of culture were filtered, 0.2 μm pore size, and the supernatant was analyzed with a Skalar Formacs TOC/TN analyzer using a Skalar LAS-160 autosampler. DIC concentrations were quantified by correlating peak area to a standard curve constructed from bicarbonate and carbonate mixtures (Sigma-Aldrich).

### Chlorophyll measurements

Chlorophylls a and b, and carotenoids were determined using the methanol extraction and optical absorption correlation described in [52]. Of the culture, 1 mL was centrifuged at 16,000×*g* for 5 min, after which the supernatant was discarded. Methanol, 1 mL, was added to the pellet, and the tube was vortexed and lightly sonicated to disperse the pellet. The suspension was heated at 70 °C for 5 min in a water bath followed by centrifugation at 16,000×*g* for 3 min. Absorption was read at 666, 653, and 470 nm. Calculations for chlorophyll and carotenoids (μg mL^−1^) were conducted as previously described in [52].

### Harvesting

Cultures were harvested just prior to medium nitrogen depletion and at the conclusion of each experiment. Each time, two aliquots of 50 mL were dispensed into 50-mL centrifuge tubes (Fisher Scientific, Palatine, IL) and centrifuged (Thermo Scientific, Sorvall Legend XTR, Waltham, MA) at 4800×*g* at 4 °C for 10 min. The concentrated biomass was rinsed with diH_2_O to remove media salts and excess bicarbonate, before centrifuging again. The remaining algal pellets were rapidly frozen and lyophilized (Labconco lyophilizer, Kansas City, MO) for 48 h and stored at −20 °C for subsequent lipid analysis.

### Extraction of lipids from dry biomass using bead beating

Extraction and analysis of extractable lipids were conducted as previously reported [39]. Approximately 30 mg of dried biomass was combined with 1 mL of chloroform in a 1.5-mL stainless steel microvial with a silicone cap (BioSpec Products, Bartlesville, OK). Three types of beads (0.6 g of 0.1 mm zirconium/silica beads, 0.4 g of 1.0 mm glass beads, two 2.7 mm glass beads) were added to each vial before capping. A FastPrep bead beater (Bio101/Thermo Savant, Carlsbad, CA) was used to agitate the vials for six 20 s pulses at power level 6.5 followed by a 1 min cool down period between pulses. Total bead beating time was 2 min. The mixture of solvent, residual biomass, and beads was then transferred to a 15-mL Pyrex test tube with a Teflon-lined screw cap (Kimble-Chase, Vineland, NJ), and the steel microvial was rinsed twice with 1 mL of chloroform, which was also added to the test tube, bringing the total solvent volume to 3 mL. Of 15 % NaCl (*v*/*v*), 1 mL was added to enhance phase separation. Samples were then centrifuged at 1200×*g* for 2 min to separate the residual biomass. Of the organic phase, 1 mL was removed from the bottom of the test tube using a glass syringe and transferred to a 2-mL GC vial for GC-FID analysis.

### Transesterification for FAME analysis

Transesterification of fatty acids was conducted using a previously described protocol [53] with modifications. FAME composition was analyzed using gas chromatography-mass spectroscopy (GC-MS) detection. Briefly, approximately 20 mg of dried algal biomass was transferred to a 15-mL Pyrex test tube with a Teflon-lined screw cap (Kimble-Chase, Vineland, NJ). Toluene, 1 mL, and sodium methoxide, 2 mL (Fisher Scientific, Pittsburgh PA), were added to each test tube along with 10 μL of a 10 mg mL^−1^ standard mixture (C11:0 and C17:0 TAG) to monitor transesterification efficiency of the TAG into FAME. Samples were heated in an oven for 30 min at 90 °C and vortexed every 10 min. Samples were allowed to cool to room temperature before 2 mL of 14 % boron tri-fluoride in methanol (Sigma-Aldrich, St. Louis, MO) were added and samples were heated again for an additional 30 min. Samples were again allowed to cool before 10 μL of a 10 mg mL^−1^ of C23:0 FAME was added to assess the completeness of partitioning FAME into the organic phase. Additionally, 0.8 mL of hexane and 0.8 mL of a saturated salt water solution (NaCl in diH_2_O) were added. Samples were heated for 10 min at 90 °C to facilitate FAME partitioning into the organic phase, vortexed for 10 s and centrifuged at 1200×*g* for 2 min to enhance phase separation. Of the organic phase, 1 mL was removed from the top layer using a glass syringe and transferred to a 2-mL GC vial for GC-MS analysis.

### Lipid analysis

#### GC-FID

GC-FID analysis was performed according to a previously published protocol [39]. Briefly, a 1-μL splitless injection was performed using an autosampler into a GC-FID (Agilent 6890 N, Santa Clara CA) equipped with a 15-m (fused silica) RTX biodiesel column (Restek, Bellefonte PA). The initial column temperature was held at 100 °C for 1 min, before being increased to 370 °C at a rate of 10 °C min^−1^. The injector temperature was held constant at 320 °C. Helium was used as the carrier gas and column flow was held at 1.3 mL min^−1^ for 22 min, increased to 1.5 mL min^−1^, held for 2 min, increased to 1.7 mL min^−1^, and held for 12 min. All flow rate increases were set to 0.2 mL min^−2^. Calibration curves were constructed for each of the following standards: C12:0, C14:0, C16:0, C18:0, and C20:0 FFAs; C12:0, C14:0, C16:0, and C18:0 MAGs; C12:0, C14:0, C16:0, and C18:0 DAGs; along with C11:0, C12:0, C14:0, C16:0, C17:0, C18:0, and C20:0 TAGs (Sigma-Aldrich, St. Louis MO) for quantification (*r*^2^ > 0.99). This GC method allows for an estimate of the amounts of FFA, MAG, DAG, and TAGs in a single analysis as detailed in [39].

### GC–MS

GC–MS analysis was performed as follows. Briefly, 1-μL split (2:1) injections were performed using an autosampler into a GC–MS (Agilent 6890 N GC and Agilent 5973 Networked MS) equipped with a 60 m × 0.25 mm × 0.15 mm Agilent DB-23 column (0.25 μm phase thickness). The injector temperature was 280 °C, and the detector temperature was 150 °C. The initial column temperature was 50 °C (held for 1 min) and was increased to 175 °C at a rate of 25 °C min^−1^, immediately followed by a ramp at 4 °C min^−1^ to a final temperature of 230 °C which was held for 10 min before run termination. Helium was used as the carrier gas and column flow was held at 0.5 mL min^−1^. Quantities of FAMEs were determined by quantifying each response peak with the nearest eluting calibration standard based on retention time, using MSD ChemStation software (Ver. D.02.00.275), with additional analyses performed using a custom program described below. A 28-component fatty acid methyl ester standard prepared in methylene chloride (NLEA FAME mixture; Restek, Bellefonte, PA) was used for GC-MS retention time identification and response curve generation (*r*^2^ > 0.99).

### Lipid quantification and analysis

Lipid quantification was performed using a custom program developed specifically for this purpose [39]. Chromatogram data from the GC-FID and GC-MS were exported from the instrument software (Chemstation Ver. B.02.01-SR1) as a Microsoft Excel spreadsheet. The spreadsheet was then imported into a web-based software application written in Microsoft’s ASP.NET framework using C# and MVC as the coding language and paradigm, respectively. Data were stored with unique identifiers in a Microsoft SQL database. Six-point calibration curves were constructed using the LINEST function which is built into the ASP.NET framework library. Chromatogram peaks were quantified based on the closest standard, as determined by retention time of the sample peak and standard peak, respectively. All chromatograms were manually integrated and inspected prior to export to ensure accuracy and reliability. Manual calculations were performed periodically using Excel to verify software results. Lipid extraction and analysis techniques used for this work have consistently found the analytical error to be within ±1–3 % [39, 54].

### Calculations

Specific growth rates were calculated using the following equation:

$$ \mu = \ln \left(\frac{\mathrm{mt}2}{\mathrm{mt}1}\right)/\left(t2-t1\right)t2>t1 $$

Productivity was calculated using the following equation:

$$ p=\left(\mathrm{m}\mathrm{t}2-\mathrm{m}\mathrm{t}1\right)/\left(t2-t1\right)t2>t1 $$

where mt1 and mt2 are the biomass concentrations at the different time points (*t*1 and *t*2), respectively.

## Additional file

Additional file 1:
**Cell concentration of cultures of** ***C. vulgaris*** **cultured under various inorganic carbon regimes.** Description of data: final cell concentrations observed in cultures with different bicarbonate salts: (■) 0 mM HCO_3_^−^ (control), (●) 50 mM ACS grade NaHCO_3_ (grade 1), (▲) 50 mM industrial grade NaHCO_3_ (grade 2), (▼) 50 mM KHCO_3_, (♦) 50 mM NH_4_HCO_3_, (◄) 50 mM Na_2_CO_3_, and (►) 25 mM NaHCO_3_∙Na_2_CO_3_ (25 mM of sesquicarbonate was used to provide equimolar carbon) (*n* = 3).

## Abbreviations

FFA: free fatty acid
MAG: monoacylglyceride
DAG: diacylglyceride
TAG: triacylglyceride
FAME: fatty acid methyl ester
DIC: dissolved inorganic carbon
GC-FID: gas chromatography-flame ionization detection
GC-MS: gas chromatography-mass spectroscopy
Chl a: chlorophyll a
Chl b: chlorophyll b
CCM: carbon concentrating mechanism
RuBisCO: ribulose-1,5-bisphospate carboxylase oxygenase
ACS: American Chemical Society
DCW: dry cell weight
ppm: parts per million
TOC/TN: total organic carbon/total nitrogen
L/D cycle: light/dark cycle
