# Supplementary figures and images for: Optimized inorganic carbon regime for enhanced growth and lipid accumulation in Chlorella vulgaris
Source: Biotechnol Biofuels. 2015 Jun 11;8:82. doi: 10.1186/s13068-015-0265-4 (PMC4476231; doi:10.1186/s13068-015-0265-4)

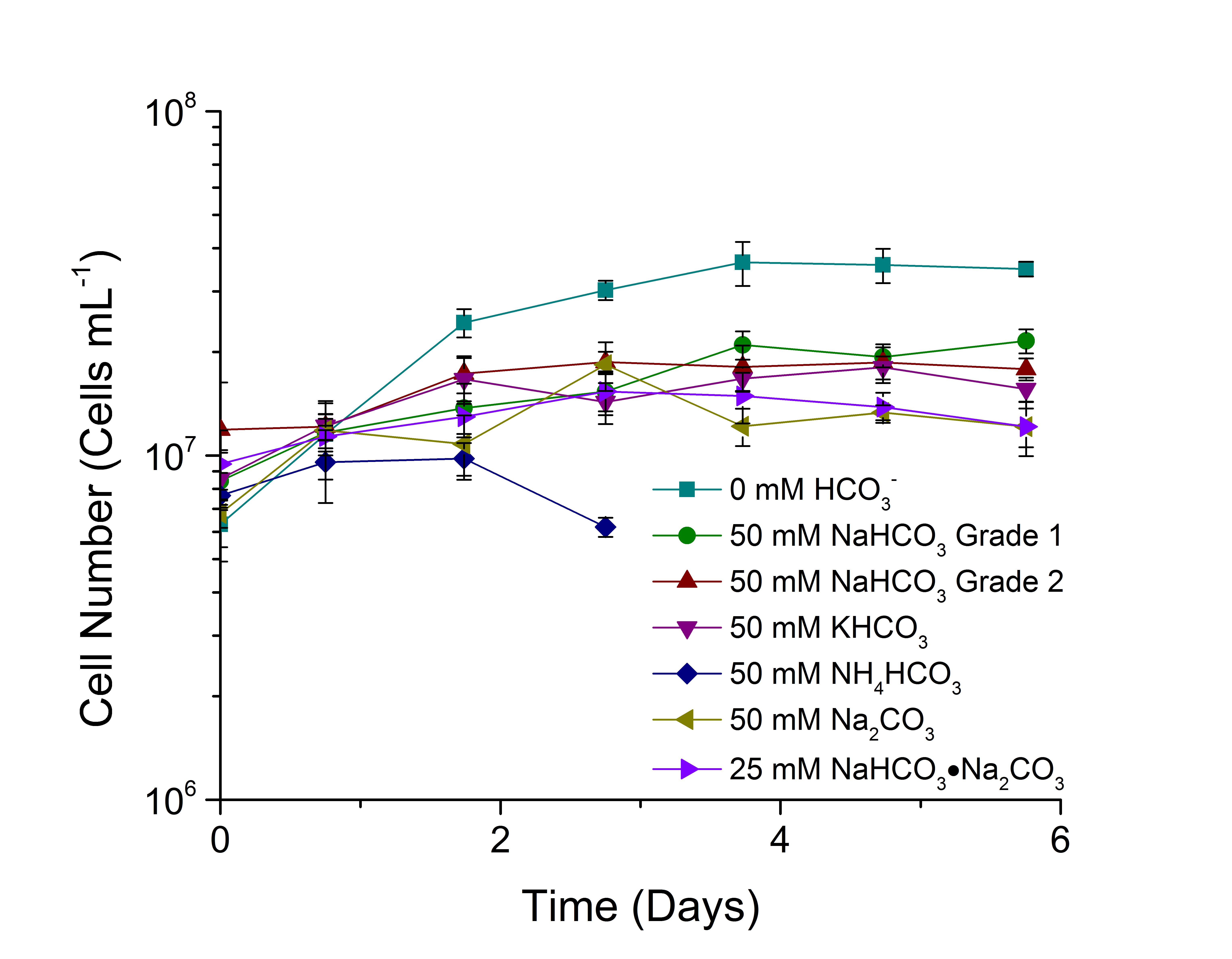

Supplement: Additional file 1: — Cell concentration of cultures of C. vulgaris cultured under various inorganic carbon regimes. Description of data: final cell concentrations observed in cultures with different bicarbonate salts: (■) 0 mM HCO3 − (control), (●) 50 mM ACS grade NaHCO3 (grade 1), (▲) 50 mM industrial grade NaHCO3 (grade 2), (▼) 50 mM KHCO3, (♦) 50 mM NH4HCO3, (◄) 50 mM Na2CO3, and (►) 25 mM NaHCO3∙Na2CO3 (25 mM of sesquicarbonate was used to provide equimolar carbon) (n = 3). [file 13068_2015_265_MOESM1_ESM.jpeg]
